# Evaluation of Different Phenotypic Techniques for the Detection of Slime Produced by Bacteria Isolated from Clinical Specimens

**DOI:** 10.7759/cureus.505

**Published:** 2016-02-23

**Authors:** Rajkumar HRV, Ramakrishna Devaki, Venkataramana Kandi

**Affiliations:** 1 Department of Microbiology, Kamineni Academy of Medical Sciences & Research Centre; 2 Department of Biochemistry, Kamineni Academy of Medical Sciences & Research Centre; 3 Department of Microbiology, Prathima Institute of Medical Sciences

**Keywords:** slime, bio-film, christensen's method, dye-elution technique, latex agglutination, congo red agar method

## Abstract

**Introduction:**

Microorganisms use various strategies for their survival in both the environment and in humans. Slime production by bacteria is one such mechanism by which microbes colonize on the indwelling prosthetic devices and form biofilms. Infections caused by such microorganisms are difficult to treat as the biofilm acts as a shield and protects microbes against antimicrobial agents. There are several methods for the detection of slime produced by bacteria, and they include both phenotypic and molecular methods. The present study evaluated the Congo red agar/broth method, Christensen’s method, dye elution technique, and the latex agglutination method for the demonstration of slime production by different bacterial clinical isolates.

**Materials & Methods:**

We collected 151 bacterial clinical isolates (both gram-positive and gram-negative bacteria) from various specimens and tested them for the production of slime both by qualitative and quantitative tests. Congo red agar/broth method, Christensen's method, dye elution technique, and latex agglutination methods were used for detecting the slime or slime-like substance.

**Results:**

We found that 103 (68.2%) strains were positive for slime production by Congo red agar/broth method. It was found that 18 (94.7%) strains of *Klebsiella* *pneumoniae*, 21 (84.0%) strains of *S aureus* and 25 (65.7%) strains of coagulase-negative *Staphylococci* were positive for slime or slime-like substances by Congo red agar/broth method. A total of 41.0% of the strains positive by Christensen's method and 15.2% of the strains by dye elution technique were found to be more adherent organisms and that have the potential to form biofilms. Only the gram-positive organisms showed nonspecific agglutination with latex suspension.

**Conclusion:**

Among the various phenotypic methods compared in this study the Congo red agar/broth method is a simple, economical, sensitive, and specific method that can be used by clinical microbiology laboratories for screening the strains for the presence of slime or slime-like substances.

## Introduction

Microorganisms have evolved during the years and found mechanisms to survive both in the environment and in humans resulting in infections [1]. Among the various survival strategies employed by microbes, the production of slime and the development of biofilms to colonize and cause serious infections assume greater significance. The introduction of devices made of biopolymers such as polycarbonate, polypropylene or polystyrene, and latex, which are highly economical and readily available under sterile conditions has revolutionized the modern management of many diseases. These biopolymers, which are extensively used in the form of several types of indwelling medical devices, became niches for many microorganisms creating a new health problem [2-4]. The organisms, which were till recently considered as laboratory contaminants and nonpathogenic, particularly the coagulase-negative *Staphylococci* are capable of adhering to these indwelling medical devices causing serious infections [5-8].

Several factors that favour the adherence of microorganisms to these devices are described and the most important among them is the production of a surface slime layer by some microorganisms that also protects the microorganism from phagocytosis and the antimicrobial action of antibiotics. The slime allows the bacteria to form biofilms—a complex matrix-like substance containing the slime (polysaccharide), the extracellular nucleic acid, and proteins—inside the human body [9-10]. So, the detection of slime produced by microorganisms can be used as a useful indicator to determine the pathogenic potential of microorganisms, their degree of virulence, and their drug resistance. [11-14]. Recent research has evaluated the correlation between biofilm production and antimicrobial resistance [15-18]. Another recent study has noted that the biofilm-producing microbes isolated from indwelling devices were showing higher resistance patterns as compared to non biofilm-producing microorganisms [19]. Few methods are described in literature for the detection of slime production by microorganisms in vitro [20-22]. Till now these methods have been mainly evaluated to detect slime production among coagulase-negative *Staphylococci *and *Staphylococcus* *aureus*. Only scanty literature is available with respect to the detection of slime and application of these methods to the gram-negative organisms, which are also very well known to produce slime [23-26]. Detection of icaA and icaD genes using polymerase chain reaction (PCR) showed that slime-producing *Staphylococci* were more virulent [27-29].

With this background we decided to take upon a study to compare the three methods i.e., Congo red agar/broth method, Christensen's method, and dye elution technique for detecting the slime or slime-like substance production by both gram-positive and gram-negative bacteria and also to study nonspecific binding of these organisms to carboxylated latex particles (Normal latex particle suspension without any antibody coating supplied by Tulip Diagnostics).

## Materials and methods

### Collection of specimens

The clinical specimens were obtained from both outpatients and inpatients attending the various departments of Kamineni Hospitals Ltd, Hyderabad. The isolates from such specimens formed the study material. All the isolates identified by colony morphology, Gram staining, and biochemical reactions were stabbed into semisolid nutrient agar medium and stored for further testing [30-31]. The following tests were conducted on these isolates.

### Congo red agar and Congo red broth method

The Congo red agar was prepared by the method described by Freeman et al., and the Congo red broth was also prepared in the same way but without the addition of the agar [20]. The advantage with the broth method is that only 3 ml of broth is sufficient for testing compared to a minimum of 20 ml of agar medium required in the plate method to test one isolate. After inoculating both the tubes and the plates with the isolates, they were incubated at 37°C under 10% CO_2_ tension and the results were noted after overnight incubation. Black color colonies on the plates and the tubes showing black* *colour was taken as positive result. The plates showing pink color colonies and the tubes showing red color were taken as negative for slime production. The controls included in the study were *Staphylococcus aureus* (ATCC 25923), *Escherichia coli* (ATCC 25922) and *Pseudomonas aeruginosa* (ATCC 27853).

### Modified Christensen’s method for quantification of slime

Polystyrene plastic tubes plugged with cotton were subjected to ethylene oxide (EO) sterilization, and sterile brain-heart infusion broth was aseptically poured (5 ml) into these tubes and checked for sterility by incubation at 37°C overnight. The isolates to be tested were inoculated into these tubes and incubated at 37°C for 24 hours. After aspirating all the contents of the tube without leaving any droplets hanging on the inner walls, the tube was gently washed with sterile water. Then a drop of 1% crystal violet stain was added and the tube was rotated thoroughly. After allowing the stain to stand for five to seven minutes, the stain was aspirated and the tube was gently washed again with sterile water to remove the excess stain. Then 0.5 ml of ethanol was added to the tube, after which the tube was swirled around for proper decolorization or elution of stain into alcohol. The intensity of the color of the eluent was then measured using a spectrophotometer at 546 nm and the optical density (OD) values were recorded. The isolates showing an OD value of more than (Mean OD + 2SD) were considered as strongly positive (more adherent) for slime production [21-22].

### Dye elution technique

Intravenous infusion sets (made of polycarbonate) were cut into 3 cm pieces and were placed in plastic tubes made of polystyrene. After they were plugged with cotton, the tubes were subjected to EO sterilization. Brain-heart infusion broth was then added and inoculation of the organisms was done in the same way as described above. After incubation overnight a few drops of 1% formalin was added to the tubes and allowed to stand for 15–20 minutes. The cut piece was removed, washed gently with distilled water and was stained completely with 1% crystal violet stain for two to three minutes. The piece was then gently washed with distilled water to remove the excess stain, after which it was placed in another tube containing 0.5 ml of ethanol and swirled/rocked gently until the piece was decolorized. Then the piece was removed. The OD values obtained were recorded and the intensity of the color that developed was measured using a spectrophotometer at 546 nm. The interpretation was made in the same way as described above [21-22].

### Latex agglutination method

An uncoated and carboxylated latex particles suspension was used as supplied by Tulip Diagnostics (P) Ltd, Goa, India. A uniform suspension of the test organism was prepared on a glass slide using sterile normal saline and a loopful of latex suspension was added. After the contents were mixed well, the slide was rocked for two minutes to observe any visible clumping. In case of ambiguity the slides were observed under a light microscope. The degree of clumping was noted as follows: no clumping (0), fine granular appearance (1+), presence of small clumps (2+), presence of large clumps (3+).

## Results

A total of 151 strains obtained from various specimens were tested for the production of slime or slime-like substances using three different techniques including Congo red agar/broth method, Christensen's method, and dye elution technique. A total of 103 (68.2%) strains were positive for slime production by both agar and broth methods. It could be seen that isolates from all the specimens were positive for slime production, the highest being 78.9% from healthy carriers of *Staphylococci, *followed by 71.4%, 71.0%, 66.6%, 64.2%, and 60.0% from urine, pus, throat swab, fluids, and blood specimens. The specimen-wise distribution of strains and positivity for slime production by different methods is elaborated in Table 1.

**Table 1:** Specimen-wise distribution of strains considered as positive (more adherent) for slime by Christensen's method (CM), dye elution technique, and Congo red agar/broth method.

Specimen	Number of Strains	Christensen’s Method n (%)	Dye Elution Technique n (%)	Congo Red Agar/Broth Method n (%)
Urine	56	19 (33.9)	08 (14.3)	40 (71.4)
Pus	38	23 (60.5)	04 (10.5)	27 (71)
Blood	15	07 (46.6)	06 (40)	09 (60)
Fluids	14	03 (21.4)	NR	09 (64.2)
Sputum	6	NR	NR	01 (16.6)
Throat swab	3	NR	NR	02 (66.6)
Skin/nasal swab	19	10 (52.6)	05 (26.3)	15 (78.9)
Total	151	62 (41)	23 (15.2)	103 (68.2)

Most of the strains showed some amount of slime production but only those showing a value of more than (Mean OD + 2SD) were included. This technique was used to classify strains as strongly positive (more adherent) and weakly positive (less adherent). A total of 41.0% of the strains positive by Christensen's method and 15.2% of the strains by dye elution technique were found to be more adherent organisms that have the potential to form biofilms.

The organism-wise evaluation of different methods for the detection of slime production is detailed in Table 2.

**Table 2:** Organism-wise evaluation of different methods for the detection of slime.

Organism (n)	Positive by Christensen's Method n (%)	Positive by Dye Elution Method n (%)	Positive by Congo Red Agar/Broth Method n (%)	Positive by Latex Agglutination Method n (%)
Coagulase negative *Staphylococci* (38)	23 (60.5)	05 (13.1)	25 (65.7)	13 (34.2)
*S aureus* (25)	17 (68)	04 (16)	21 (84)	11 (40)
*Klebsiella pneumoniae* (19)	07 (36.8)	05 (26.3)	18 (94.7)	NR
*E coli* (50)	05 (10)	07 (14)	39 (78)	NR
*Pseudomonas aeruginosa* (19)	10 (52.6)	02 (10.5)	NR	NR
Total (151)	62 (41)	23 (15.2)	103 (68.2)	24 (15.8)

It was seen that 18 (94.7%) strains of *Klebsiella* *pneumoniae*, 21 (84.0%) strains of *S aureus* and 25 (65.7%) strains of coagulase-negative *Staphylococci* were positive for slime or slime-like substances by Congo red agar/broth method. None of the strains of *Pseudomonas* *aeruginosa* were positive by this method. However, 17 (68.0%) strains of *S aureus*, 23 (60.5%) strains of coagulase-negative *Staphylococci* and 10 (52.6%) strains of *Pseudomonas* *aeruginosa* were found to be strongly positive (more adhering) for slime by Christensen's method. By dye elution technique comparatively fewer number of strains were more adherent. It was seen that only the gram-positive organisms showed nonspecific agglutination with latex suspension.

## Discussion

The introduction of indwelling medical devices made of biopolymers like polystyrene, polypropylene, silicone, rubber, latex, etc., has revolutionized health care and management of patients with various diseases. However, the extensive usage of these prosthetic devices made of biopolymers has resulted in a new public health problem, namely biofilms and device-associated infections. Among various mechanisms used by microbes to establish themselves in a human body and cause infection, the formation of biofilms assumes greater significance [32-33]. Biofilm formation allows bacteria to colonize and cause chronic infections that are difficult to treat [34-35]. Recent research emphasizes the significance of antibiotic-induced biofilm formation [36]. Research in the past has emphasized the role of biofilm in wound healing and proposed potential therapeutic solutions for infections caused by biofilm-producing microorganisms [37-40]. Previous research has also suggested that microorganisms that are able to produce slime and form biofilms have mechanisms to synthesize some special molecules that facilitate its survival in humans [41]. Most of these infections are caused by microorganisms that are considered as normal flora of the skin or oral cavity and are dismissed as contaminants. *Staphylococcus epidermidis *tops the list among the organisms capable of causing device-associated infections. Other organisms including *Staphylococcus aureus*, *Klebsiella pneumoniae*, *Escherichia* *coli*, and *Pseudomonas* *aeruginosa* have also been isolated from such infections.

From these observations, it is clear that the detection of slime-producing* Staphylococci* may be very useful for clinical decision-making, as these microbes are difficult to eradicate. Several techniques that are either qualitative or quantitative have been described for determining the adherence potential or slime-production by coagulase-negative *Staphylococci. *These include the Congo red agar method and Christensen's method. Merritt et al. described another simple technique to quantify the bacterial adherence by extracting the biofilm with ethanol, after fixing with formalin and staining with 1% crystal violet [42]. Although Christensen described a quantitative microtitre technique, most of the studies were restricted to only qualitative observation of the staining of the biofilm that is adhered to a glass or a biopolymer surface. However, no literature is available to the best of our knowledge, as to which of these techniques could be applied for detection of biofilm formation in the case of gram-negative bacteria.

Keeping these observations in view, we simultaneously tested for slime-production by the Congo red agar/broth method and quantified it by Christensen's method and the dye elution technique. Among the strains tested by Congo red broth method, a total of 103 (68.2%) strains were positive for slime production. All these strains were also positive when tested with Congo red agar method showing that the Congo red broth method is equally effective as the agar method for slime detection.

Among the gram-positive bacteria both *S aureus* and coagulase-negative* Staphylococci*  showed 84% and 67% positivity with Congo red broth test. Nayak et al. studied 176 strains of coagulase-negative *Staphylococci* and found that 99 (56.3%) were slime positive. In their study slime-positive strains were isolated in significantly higher number from patients than controls. The same study also has noted that 54 (22.1%) strains, which were positive by Congo red agar method for slime, were negative by Christensen's method. So they concluded that Congo red agar method is a more sensitive and better screening method for slime detection when compared to Christensen's method [21].

In the present study, among gram-negative bacteria *K pneumoniae* showed slime production in 94.7% of cases followed by *Escherichia* *coli* in 78% of cases. None of the *Pseudomonas* *aeruginosa* strains tested showed positivity by Congo red broth method. The results suggest that the Congo red test may not be useful for identifying the exopolysaccharide layer-producing nonfermenting gram-negative bacteria, especially *Pseudomonas* spp. This is because these bacteria do not ferment any other sugars that may be necessary to release certain metabolites, which combine with Congo red to impart a black colour to the colonies indicating slime production. Not many studies are available in literature to the best of our knowledge to compare our findings. In Christensen's method 60.5% of coagulase-negative *Staphylococci* and 68% of *S aureus* were strongly positive for slime (i.e., more adherent) followed by 13.1% and 16% by dye elution technique. Gram-negative bacteria showed more adherences in comparatively fewer number of cases except for *Pseudomonas* *aeruginosa* which showed more adherence in 52.6% of cases by Christensen's method. Makhija et al. studied Christensen's method and found that 42.5% strains of coagulase-negative *Staphylococci* were slime-positive [43].

In Christensen's method that was used to quantify slime, 41% of the total number tested (both gram-positive and gram-negative isolates) were strongly positive for the slime as the values were higher than (Mean OD + 2SD). Mathur et al. in their study have evaluated three different methods for the detection of slime among *Staphylococci*: tissue culture plate (TCP) method, tube method (TM), and congo red agar method. They found that the TCP method was a more sensitive, accurate, and reliable technique that can also be used as a quantitative model [6]. Knobloch et al. also evaluated biofilm formation among ica gene-positive strains of *S aureus* and found that the TCP method was the most sensitive and specific method. The Congo red agar method was found ineffective as only 3.6% strains correlated with the TCP and tube methods [44]. Another recent study that tested 70 isolates including both gram-positive and gram-negative bacteria has found that the TCP method was more sensitive (98%) than the Congo red agar method (89%). El-Khier et al. in their study evaluated the ability of the *S. epidermidis* isolated from orthopedic implants and prostheses to form biofilm. This study included both phenotypic and genotypic detection methods and found that the congo red agar method had a sensitivity of 93.3% and specificity of 87.5% while the TCP assay represented 100% sensitivity and 79.2% specificity [5]. Prasad et al. in their study evaluated biofilm activity among *Pseudomonas* *aeruginosa* and found that the tube method is more effective [45]. A study from Pakistan by Hassan et al. found the TCP method to be a more reliable quantitative method and recommended its use as a routine screening technique [46]. De Castro Melo et al. screened for biofilm formation among *S aureus* strains isolated from animals using both molecular and phenotypic methods. This study observed that both the Congo red agar and the TCP methods had 100% specificity when correlated with molecular methods [47].

The above findings clearly indicate that the Congo red test is a very sensitive method for the detection of slime or slime-like substances. It is probable that a very high concentration of sucrose (a polymer of oligosaccharides) present in Congo red broth is responsible for stimulating the production of slime, which may combine with Congo red and yield a black color. In Christensen's method only 41% of strains were more adhering followed by 15.2% of strains by dye elution technique. Although the least number of strains were positive by dye elution technique, its specificity was higher than Christensen's method. When the ethanol extract, which was quantified in terms of absorbance at 546 nm, was compared in both the methods, it was seen that OD values of dye elution technique were high compared to Christensen's method.

This phenomenon can be explained by the type of material used i.e., polystyrene tubes in Christensen's method and intra-venous infusion set material made of polycarbonate in dye elution technique. In addition this may also be due to other properties such as hydrophobicity, hydrophilic nature, and electrostatic forces, which are mediated by extracellular macromolecules because other parameters like type of media, pH, time and temperature of incubation etc, were same for both the methods. It has been noted that the strains with the greatest hydrophobicity were the most adhering to a variety of synthetic polymers.

The agglutination of gram-positive cocci with carboxylated latex suspension is an interesting phenomenon noticed in this study. A total of 24 strains of *S aureus* and coagulase-negative *Staphylococci* showed agglutination or clumping. This phenomenon was not noticed in other gram-negative bacteria tested in the study. The exact reason behind this could not be clearly explained, though theoretically, it could be due to coalescing of small molecules. Nonspecific physical properties like ionic strength and zeta potential might be playing a role and that needs to be confirmed by further studies. 

Another possibility for clumping might be the presence of amino groups (NH_2_) on the surface of gram-positive bacteria. The hydrogen atom of the carboxyl group of the latex suspension may bind to one hydrogen atom of the amino groups resulting in the formation of a peptide bond, with the release of a water molecule.

COOH + H_2_N = CO-NH + H_2_O

However, it needs to be further confirmed whether such a phenomenon really occurs in vivo. No studies are available as of now for comparing our findings with respect to the nonspecific agglutination with carboxylated latex particles.

This study clearly shows that both gram-positive and gram-negative bacteria produce slime or slime-like substances. But when quantification was done it shows that only gram-positive bacteria were`strongly positive (more adhering) for slime than gram-negative bacteria. This could be the reason why gram-positive cocci especially coagulase-negative *Staphylococci* are responsible for severe forms of device-associated infections, though gram-negative bacteria can also be associated in a few cases.

The results of the current study indicate that a great percentage of clinical isolates have potential to produce slime and in turn form biofilms. Infections caused by these microbes are difficult to treat, and therefore, effective diagnostic, therapeutic, and management strategies are warranted [48-50].

## Conclusions

In conclusion, our study highlights that the Congo red agar/broth method is a simple, economical, sensitive, and specific method that can be used by clinical microbiology laboratories for screening the strains for the presence of slime or slime-like substances. Those strains showing positive reactions can be confirmed by the quantification of slime either by Christensen's method or by dye elution technique. Between Christensen's method and dye elution technique, the latter was found to be more specific and easily adaptable to routine laboratory testing so that physicians can be alerted for necessary clinical intervention. Approximately one-third of staphylococcal strains (both coagulase-negative *Staphylococci* and *S aureus*) showed nonspecific agglutination with uncoated carboxylated latex particle suspension, which was in correlation with results obtained by the Congo red method and Christensen's method. Further studies in future are warranted as to whether the latex agglutination method can also be used as a screening test for *Staphylococci* that are positive for slime-like substance. This will further help the rapid screening of the slime-producing strains. We also observed that although slime-producing strains were sensitive to some of the antibiotics tested, patients did not show any clinical response till the devices in situ were removed, which clearly demonstrates that slime does play a role in protecting an organism against the bactericidal action of various antimicrobial agents. Although previous studies are available in literature that have compared various slime detection methods, the results are contrasting and there is still no clear consensus on which method is applicable for routine use in clinical microbiology laboratories. Molecular methods for the detection of genes coding for slime production is a gold standard. But considering the fact that it is not feasible to routinely use this procedure, evaluating various phenotypic methods and selecting the most appropriate one for regular laboratory use is important.
